# Neonatal Intrathoracic Gastric Volvulus in Marfan's Syndrome

**DOI:** 10.1055/s-0038-1666795

**Published:** 2018-07-13

**Authors:** Javier Serradilla, Alba Bueno, Carlos De La Torre, Eduardo Alonso Gamarra, Martha Muñoz Romo, Francisco de Borja Nava Hurtado de Saracho, María Álvarez Barrial, Manuel Gomez Cervantes, Manuel Lopez Santamaria

**Affiliations:** 1 Department of Pediatric Surgery, Hospital Universitario La Paz, Madrid, Spain; 2 Department of Radiology, Hospital Universitario La Paz, Madrid, Spain

**Keywords:** Marfan's syndrome, neonatal, intrathoracic stomach, hiatal hernia, gastric volvulus

## Abstract

We report a 12-day-old male who was admitted with vomiting because of an unusual early complication of Marfan's syndrome (MS): a sliding hiatal hernia. Initial ultrasound showed no stomach at its normal position and the chest X-ray presented an intrathoracic gas bubble with the nasogastric tube inside. An upper gastrointestinal contrast study confirmed the complete herniation of the stomach into the thorax. Via an exploratory laparotomy it was carefully reintroduced into the abdomen, following a hiatal reconstruction. A Thal fundoplication and a gastrostomy were also performed to guarantee its fixation. Although characterized by cardiac/aortic abnormalities, MS should be considered in any infant with hiatal/paraesophageal hernia, which should be repaired early to avoid gastric ischemia/volvulus.

## Introduction

Marfan's syndrome (MS) is a systemic connective tissue disorder which mainly involves ocular, skeletal, and cardiovascular systems. 1

Antoine Marfan described the first case in 1896 2 and its current frequency is estimated to be 1:5,000 to 1:10,000, without enrichment in any ethnic or racial group and no gender preference.

It is caused by an autosomal dominant mutation of *FBN1* with a high degree of clinical variability, ranging from isolated features to severe and rapidly progressive disease in multiple organ systems. 3

Gastrointestinal symptoms are usually rare. Most of the time, this system only gets involved by noncomplicated inguinal hernias which are the most frequently reported hernias in MS. 2 4 However, despite being really rare, hiatal hernia, paraesophageal hernia, and intrathoracic stomach have all been reported in MS and are thought to be due to the abnormality of gastric ligaments and diaphragm. 4 5 These entities become important because of their tendency to relapse and the possibility of triggering extremely rare but critical situations such as a gastric volvulus. 1 6

Various types of hernias are seen in MS, such as inguinal hernias and an occasional hiatus hernia.

Classically, MS diagnosis has been based on family history and the observation of its characteristic findings. In fact, ectopia lentis and aortic aneurysm have a special clinical significance because of their specificity and frequency. 3 7 Instead, the sensitivity of molecular genetic testing of FBN1 is substantial yet incomplete because of its large heterogeneity. 1

MS rarely presents with symptoms in the neonatal period. 2 6 Our objective is to report one of these rare cases of MS that debuted with the complete herniation of the stomach into the thorax through a large hiatal hernia.

## Clinical Report

A 12-day-old male infant was admitted with recurrent nonbilious vomiting. The patient was born to a 35-old-gravida by spontaneous vaginal delivery at 40 weeks, weighing 2,868 g, after a controlled pregnancy and a threat of preterm delivery at 30 + 6 weeks solved with tocolytic and corticoid treatment. The Appearance, Pulse, Grimace, Activity, and Respiration scores were 9 at 1 minute and 10 at 5 minutes.

**Fig. 1:**
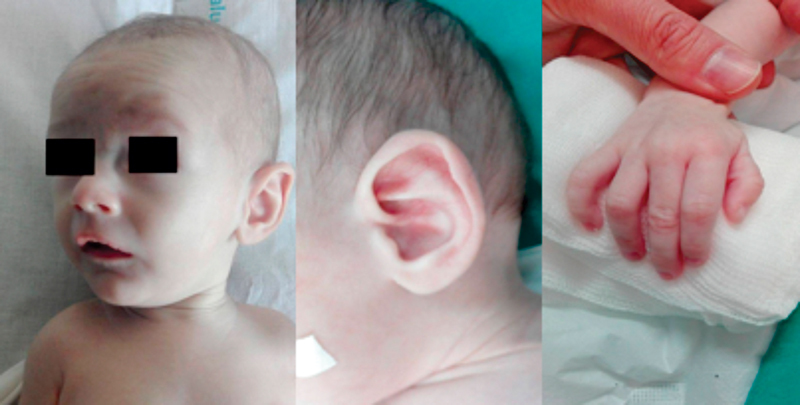
Patient's senile appearance.

The mother and the patient displayed features of MS. The patient was a small, thin infant with “senile” appearance ( Fig. 1 ), micrognathia, crumpled ears, and reduced muscle mass and subcutaneous tissue. The fingers were long, overlapping, and mildly hyperextensible and his wrists were deviated ulnarly. He showed flexion deformities with difficulty in extending the elbows, hips, and knees. Left inguinal hernia was also present.

The parents reported that the maternal grandfather had a similar phenotype but none of them had previous cardiac events.

**Fig. 2:**
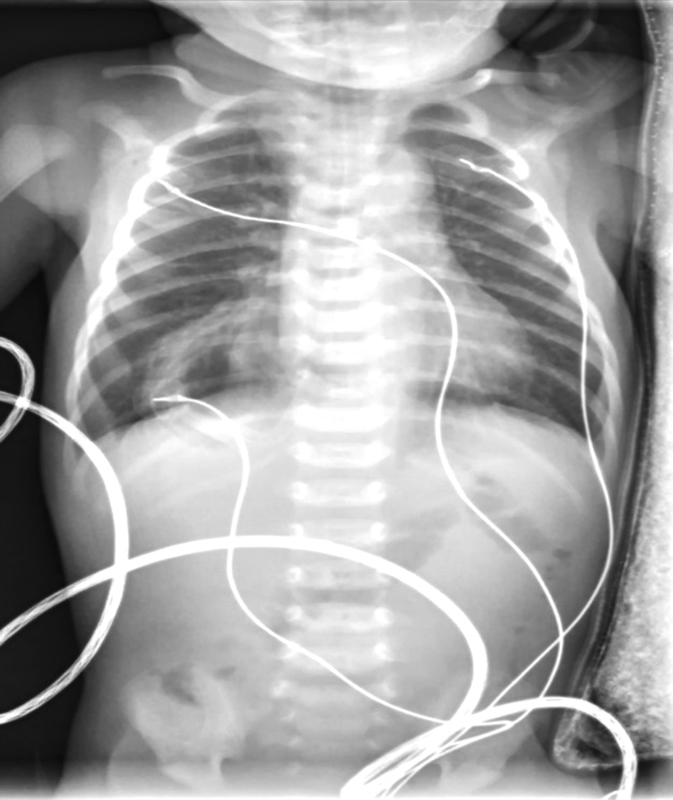
Chest X-ray showing an intrathoracic gas bubble compatible with stomach.

**Fig. 3:**
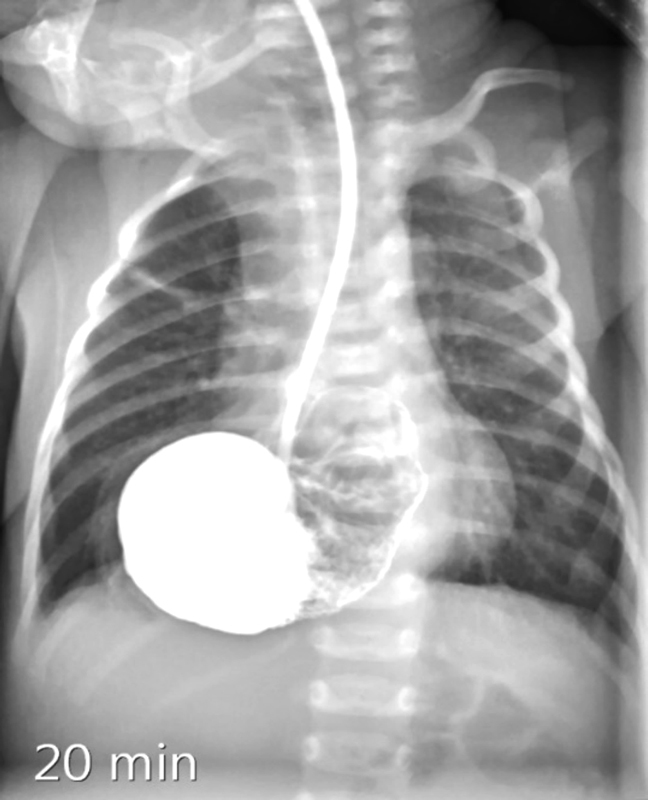
Upper gastrointestinal contrast study confirming the complete herniation of the stomach into the left thorax.

A nasogastric tube was placed, obtaining nonbile stained gastric content. Ultrasound showed no stomach at its normal position, but a compatible structure was observed through the right diaphragm and an intrathoracic gas bubble with the nasogastric tube inside was seen ( Fig. 2 ). Blood test was completely normal. The diagnostic workup completed by an upper gastrointestinal water-soluble contrast study, confirming the complete herniation of the stomach into the right thorax ( Fig. 3 ). The images evinced a partially volvulating stomach with pylorus on top and the duodenum crossing the hiatus, making the passage of the content to the intestine much more difficult.

Subsequent echocardiography documented mildly dilated ascending aorta. Ophthalmic examination was normal.

**Fig. 4:**
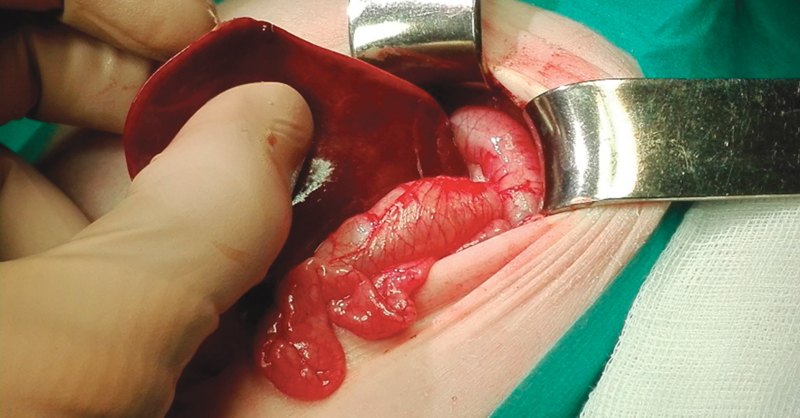
Large and partially volvulating stomach, almost completely herniated into the thorax.

**Fig. 5:**
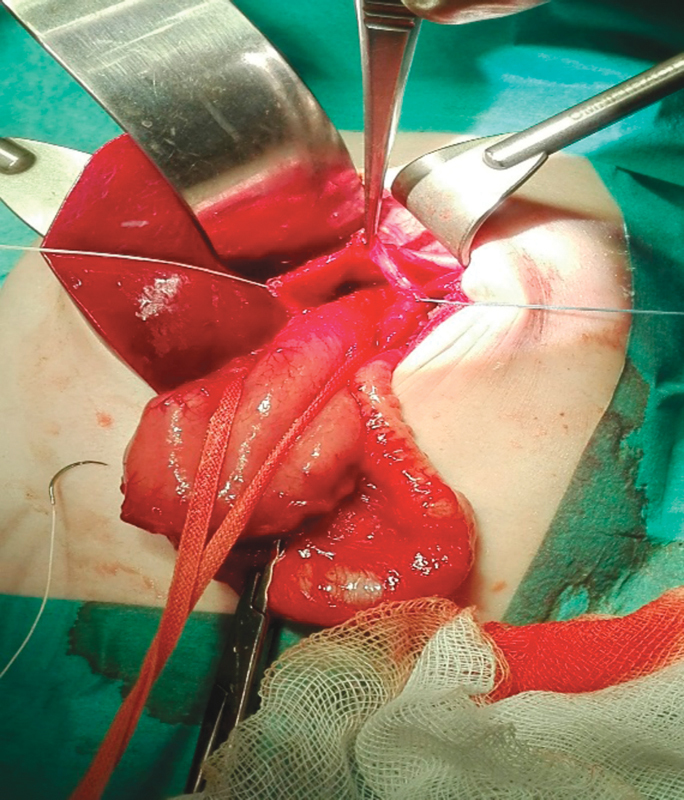
Wide diaphragmatic hiatus.

The decision for surgical exploration was made through a longitudinal supraumbilical laparotomy. We confirmed the presence of a large stomach, volvulating over its mesenteroaxial axis and almost completely herniated ( Fig. 4 ) through a wide diaphragmatic hiatus ( Fig. 5 ). The transverse colon was also forming part of the hernia. A hiatal dissection was performed after reducing the organs into the abdomen by pulling them carefully with atraumatic surgical instruments. Using a large nasogastric tube into the esophagus and stomach, the hiatus was repaired with an absorbable mesh reinforcement (Vicryl, Ethicon). A modified Thal fundoplication was performed. To guarantee the fixation of the stomach on its normal position, a gastrostomy (Stamm's technique) was realized.

After surgery, the infant was admitted to the neonatal intensive care unit. The postoperative course was uneventful. Oral feedings were commenced on the third day, being gradually advanced and tolerated. He was discharged with an adequate and exclusive oral tolerance after 7 days.

He was readmitted 3 months later for left inguinal hernia repair and gastrostomy withdrawal. During the later life, genetic consultation confirmed the diagnosis of MS in the patient and his mother.

After 2 years of follow-up, the patient remains asymptomatic maintaining an adequate oral tolerance and an appropriate weight gain.

## Discussion

Although the diagnosis of MS is more common in adolescence and adulthood because of the typical body habitus and the development of the most typical complications (mitral valve prolapse and other cardiological events), 2 it is important to know that it may also debut in the neonatal period.

We should suspect this condition in a newborn with abnormal habitus, heart valve insufficiency, cutis laxa, crumpled ears, joint contractures, muscle hypoplasia, congenital anomalies of the eye, or pulmonary hypoplasia. This might occur with a negative family history for MS. Various types of hernias are also seen in MS, such as inguinal hernias 4 and an occasional sliding hiatal hernia because of the unusual elasticity of tissues. 2 4 5 8 Lax gastric ligaments (gastrohepatic, gastrophrenic, gastrosplenic, gastrocolic) and a deficient diaphragmatic hiatus predisposes to it. 2 5 8

In this case, patients usually display symptoms of preduodenal intestinal obstruction like nonbilious vomiting 4 6 8 or respiratory distress and a Marfan's phenotype or familiar history. 5 8 We should consider this entity in patients that present these characteristics because prompt recognition can facilitate management and counseling.

After confirmation of the diagnosis, it is essential to reposition the stomach into the abdomen to avoid gastric necrosis by a complex gastric volvulus. 5 8 9

After the hiatal repair, we chose to place an absorbable mesh to induce fibrosis and therefore local reinforcement. We chose a Vicryl mesh to avoid the presence of a foreign material in the long term. Nevertheless, a funduplication and gastrostomy must be considered as a part of the surgical approach to prevent reflux and recurrence. 5 8 9

It is important to emphasize that gastrostomy is only performed to fix the stomach. If possible, it should not be used for feeding to preserve the correct development of the feeding/swallowing mechanism in the newborn. It might be withdrawn after 3 to 6 months if the patient's clinical condition allows it.

In summary, MS should be considered in any infant with hiatal/paraesophageal hernia, which should be repaired early to avoid gastric volvulus. These infants require long-term multidisciplinary follow-up to detect recurrences and other early complications of MS. 8 9 10

## Notes

New Insights and the Importance for a Pediatric Surgeon Prompt recognition of this entity can facilitate management and counseling, avoiding ischemic necrosis by a complex gastric volvulus.
